# Risk factors of early childhood caries (ECC) among children in Beijing - a prospective cohort study

**DOI:** 10.1186/s12903-019-0721-9

**Published:** 2019-02-18

**Authors:** Can Can Fan, Wen Hui Wang, Tao Xu, Shu Guo Zheng

**Affiliations:** 1 Department of Preventive Dentistry, Peking University School and Hospital of Stomatology, National Engineering Laboratory for Digital and Material Technology of Stomatology, Beijing Key Laboratory of Digital Stomatology, 22 Zhongguancun Avenue South, Haidian District, Beijing, 100081 People’s Republic of China; 2 Guanghua School of Stomatology, Guangdong Provincial Key Laboratory of Stomatology, Sun Yat-sen University, 56 Ling Yuan Road West, Guangzhou, 510055 Guangdong Province People’s Republic of China

**Keywords:** Early childhood caries, Mutans streptococci, Risk factors, Previous caries experience

## Abstract

**Background:**

Early childhood caries (ECC) was a serious oral health concern with high prevalence and a low treatment rate in China, and few researches have focused on preschool children. This study aimed to explore the risk factors of ECC in Beijing, China.

**Methods:**

Totle of 712 children (mean age: 46.37 ± 5.44 months) participated in this prospective cohort study. Questionnaires and the levels of mutans streptococci in dental plaques and non-stimulated saliva, assessed using Dentocult SM strips, were collected at baseline, respectively. Two calibrated examiners checked for dental caries according to the WHO 1997 criteria at baseline and 1-year follow-up. Negative binomial regression was used for multivariate analysis of factors related to caries development.

**Results:**

For caries-free children at baseline, only plaque mutans streptococci (PMS) levels were associated with caries development (odds ratio [OR] = 1.68, 95% confidence interval [CI]: 1.08–2.61, *P* = 0.02). Children with high PMS levels (scored 2 and 3) had higher caries incidence (46.2% vs. 33.8%) and more caries increment (1.87 ± 3.17 vs. 0.90 ± 1.89) than those with low levels (scored 0 and 1). Among all participants, PMS (OR = 1.69, 95% CI: 1.32–2.23, *P* < 0.001) and previous caries experience (OR = 2.80, 95% CI: 2.20–3.56, *P* < 0.001) were related to caries increment.

**Conclusions:**

For caries-free children, the only significant risk factor for ECC was high PMS levels. For all participants, besides high PMS levels, previous caries experience was another significant risk factor for caries development.

**Trial registration:**

The study design, protocol, and informed consent forms were undertaken with the understanding of Declaration of Helsinki and were approved by the Human Research Ethics Committee of the School of Stomatology, Peking University, China (PKUSSIRB-2012042).

## Background

Early childhood caries (ECC) is an urgent concern in China with high prevalence and a low treatment rate. The prevalence of dental caries and the mean decayed, missing, and filled teeth (dmft) score for 5-year-old children in Beijing were 58.6% and 2.57, in 2005 [1]. However, after 5 years, these two indexes were found increased to 65.5% and 3.26, in the same age group of children, respectively [2]. ECC has shown a marked increase in several areas of China during the past decade [3]. However, studies focusing on ECC were rare; in particular, longitudinal studies on factors related to ECC are lacking in Beijing and/or in China.

Caries distribution among children is polarized, with 75% of affected tooth surfaces being reported in < 25% of children [4, 5]. If the risk factors for development of ECC in children were identified and timely prevention are provided for the high-risk children, a multiplier effect could be achieved. The commonly accepted risk factors for dental caries include dietary habits (e.g., sugary snacks), poor oral hygiene, microbiological factors, and low socioeconomic status [6, 7]. According to available evidence, the risk factors for caries varies among children with different backgrounds, and are also affected by the study designs, participants, and statistical analysis techniques used [8, 9]. Several studies have recognized the importance of infection of mutans streptococci [10]. Although the levels of mutans streptococci are a strong risk indicator for ECC, additional well-designed longitudinal studies with high evidence levels are warranted to confirm the level of mutans streptococci as a significant ECC risk factor in China.

There are many testing methods are used to detect pathogen levels in the oral cavity. The Dentocult SM strip mutans test (Orion Diagnostica, Espoo, Finland) has been used in several relevant ECC studies [11, 12]. It is a simple test kit and provides a convenient and practical semiquantitative method to detect the levels of mutans streptococci among infected individuals [13]. The aim of this prospective cohort study was to investigate factors associated with the development of ECC in Beijing, China, particularly among children who are caries free at baseline, and to investigate whether the levels of mutans streptococci in both dental plaque and saliva could be a significant risk factor for ECC with the use of Dentocult SM strips.

## Material and methods

### Ethics statement

Written informed consent was obtained from the guardians of all children included in this study. The study design, protocol, and informed consent forms were undertaken with the understanding of Declaration of Helsinki and were approved by the Human Research Ethics Committee of the School of Stomatology, Peking University, China (PKUSSIRB-2012042).

### Participants

This study was the longitudinal part of a previous published research in Haidian District of Beijing, where the fluoride concentration in the drinking water is around 0.3–0.4 mg/L. Water fluoridation, salt fluoridation and other systemic use of fluoride are not available in China. Topical use of 1.23% fluoride foam is implemented twice per year among all preschool children in kindergartens in Beijing. A convenience sampling method was used, six cooperative kindergartens near our hospital were selected for study: a cohort of 787 children aged 3–4 years were recruited at baseline. Details of sampling method have been described in a previously published article [14]. The dental treatment for all participants with caries after baseline examination were not interfered.

### Questionnaire

At baseline, the guardians were asked to provide detailed information about their children’s demographic information (gender, birth date), dietary habits (using nursing bottle or not and the content, frequency of sucrose diet), oral hygiene practices (brushing frequency, guardians’ help, toothpaste containing fluoride or not), dental visit history. And the guardians’ educational levels were also obtained through the questionnaire.

### Microbiological test

The levels of mutans streptococci in both non-stimulated saliva and dental plaque were evaluated with Dentocult SM Strip (Orion Diagnostica, Espoo, Finland) at baseline. No paraffin was used for salivary stimulation. The saliva samples were obtained by pressing the rough surface of the strip against the child’s tongue and turning it over 10 times. Four specific sites of supragingival dental plaque including the buccal surfaces of teeth 55, 51, and 71, and the lingual surface of tooth 75 were sampled. Plaque samples were collected by stroking the tooth surface near the gingival margin with a separate mini-brush for each tooth, and applied to the four roughened sites on the strip, and the strips were then placed in the culture medium and incubated at 37 °C for 48 h.

One experienced examiner who were blinded to the subject’s caries status assessed the levels of mutans streptococci by the counts of colony forming units per milliliter (CFU/ml) according to the manufacturer’s instruction. The score “0” corresponds to < 10^4^ of CFU/mL, “1” to 10^4^–10^5^ of CFU/mL, “2” to 10^5^–10^6^ of CFU/mL, and “3” to > 10^6^ of CFU/mL. The plaque mutans streptococci level of the child was the highest score of the four sites.

### Dental examination

All children were examined at baseline and 1-year follow-up by two calibrated examiners who were blinded to the children’s microbiological test results. The CPI (community periodontal index) explorer and disposable mirror were used to diagnose dental caries at the tooth surface level, with a cotton swab to dry the teeth. The diagnostic criteria were according to the WHO 1997 criteria by visual inspection, applied with tactile inspection if necessary. No radiographs were taken. Before this investigation, the two examiners conducted an examination of fifteen subjects and the inter-examiner kappa value was 0.82. Fifteen children were reexamined by the two examiners one week later for the calculation of intra-examiner kappa values, and the values for the two examiners were 0.88 and 0.86.

### Statistical analyses

Data analysis was performed for children who completed questionnaires, dental examination, and assessment of mutans streptococci levels in dental plaque and non-stimulated saliva at baseline as well as 1-year follow-up tooth decay check-up. Caries incidence was defined as the presence of at least one new carious surface at 1- year follow-up examination. The chi-square test was used in univariate analyses to assess the differences between children with or without new caries. The increase in decayed, missing, and filled surfaces (dmfs) scores at 1-year follow-up of children with different levels of mutans streptococci infection were calculated and compared using *t* test with SPSS Statistics for Windows (version 20.0; IBM Corp. Armonk, NY, USA). Negative binomial regression was used to identify the variables associated with caries incidence and caries increment; the SAS software (version 9.3; SAS Institute, Cary, NC, USA) was used for this analysis. Statistical significance was set at *P* < 0.05.

## Results

### General information

Of the 787 children who answered the questionnaires and participated in the dental examination and lab test at baseline, 75 children were lost to follow-up at 1 year after baseline examination, primarily because they left the schools or did not attend the follow-up examination. Finally, 712 (90.5%) subjects remained in the study (mean age: 46.37 ± 5.44 months at baseline). Among these 712 children, 361 children were caries free at baseline, which we called the caries-free group. The following results of univariate and multivariate analyses were reported for children in the caries-free group and all participants respectively, the general information of two groups was presented in Table 1.

**Table 1:** Baseline characteristics of participants and 1-year caries incidence in the CF group and all children

Variables	CF group		*P*	All participants		*P*
	Without new caries N (%)	With new caries N (%)		Without new caries N (%)	With new caries N (%)	
Age (months) at baseline	45.8 ± 5.5	45.7 ± 6.1	0.96	45.8 ± 5.4	46.7 ± 5.5	0.68
Gender			0.91			0.75
Male	110 (49.3)	67 (48.6)		138 (51.9)	216 (53.3)	
Female	113 (50.7)	71 (51.4)		128 (48.1)	189 (46.7)	
Person filling out the questionnaire			0.63			0.80
Father	52 (24.1)	29 (21.3)		61 (23.6)	99 (25.3)	
Mother	142 (65.7)	93 (68.4)		170 (65.6)	249 (63.7)	
Grandfather/Grandmother	20 (9.3)	14 (10.3)		26 (10.0)	38 (9.7)	
Other (relative/nanny)	2 (0.9)	0 (0.0)		2 (0.8)	5 (1.3)	
Using nursing bottle or not at present			0.22			0.01*
Yes	60 (27.8)	30 (21.9)		68 (26.3)	72 (18.4)	
No	156 (72.2)	107 (78.1)		191 (73.7)	32 (81.6)	
Contents of nursing bottle			0.11			0.11
Water or milk	77 (96.3)	59 (84.3)		90 (49.2)	162 (54.5)	
Sugared beverages	3 (3.7)	11 (15.7)		93 (50.8)	135 (45.5)	
Sleeping with nursing bottle			0.59			0.58
Always	2 (4.1)	0 (1.5)		2 (1.5)	7 (3.6)	
Sometimes	15 (12.3)	6 (11.3)		17 (12.8)	25 (12.9)	
Rarely or never	197 (83.6)	127 (87.2)		114 (85.7)	162 (83.5)	
Frequency of snack consumption			0.29			0.04*
Never or seldom	21 (9.7)	6 (4.4)		24 (9.3)	19 (4.9)	
Sometimes (< 1 Times/Day)	55 (25.4)	30 (21.9)		69 (26.6)	88 (22.6)	
1–2 Times/Day	106 (49.1)	73 (53.3)		123 (47.5)	188 (48.3)	
3–4 Times/Day	30 (13.9)	25 (18.2)		37 (14.3)	80 (20.6)	
≥ 5 Times/Day	4 (1.9)	3 (2.2)		6 (2.3)	14 (3.6)	
Rinsing or brushing teeth after snacks			0.71			0.59
Always	60 (27.8)	38 (27.7)		74 (28.6)	104 (26.7)	
Sometimes	95 (44.0)	66 (48.2)		114 (44.0)	181 (46.5)	
Never or seldom	61 (28.2)	33 (24.1)		71 (27.4)	104 (26.7)	
Sleeping without brushing after snacks			0.27			0.66
Always	15 (6.9)	5 (3.7)		16 (6.2)	18 (4.6)	
Sometimes	54 (25.0)	29 (21.3)		64 (24.7)	91 (23.5)	
Never or seldom	147 (68.1)	102 (75.0)		179 (69.1)	279 (71.9)	
Frequency of tooth brushing			0.62			0.01*
Twice daily	89 (41.2)	53 (39.6)		109 (42.1)	179 (46.3)	
Once daily	88 (40.8)	63 (47.0)		102 (39.4)	168 (43.4)	
Less than once daily	35 (16.2)	16 (11.9)		43 (16.6)	36 (9.3)	
Never	4 (1.9)	2 (1.5)		5 (1.9)	4 (1.0)	
Guardians helping with the brushing			0.63			0.62
Everyday	50 (23.3)	33 (24.3)		60 (23.3)	106 (27.3)	
Sometimes	76 (35.3)	52 (38.2)		91 (35.3)	138 (35.6)	
No or never brushing	89 (41.4)	51 (37.5)		107 (41.4)	144 (37.1)	
Toothpaste containing fluoride			0.32			0.03*
Yes	77 (36.0)	38 (28.8)		94 (36.9)	137 (35.7)	
No	65 (30.3)	47 (35.6)		77 (30.2)	127 (33.2)	
Not sure	53 (24.8)	39 (29.5)		60 (23.5)	104 (27.2)	
Not having used toothpaste	19 (8.9)	8 (6.1)		24 (9.4)	15 (3.9)	
Dental visit history			0.41			< 0.001**
Yes	63 (29.3)	46 (33.8)		91 (35.3)	238 (61.5)	
No	152 (70.7)	90 (66.2)		167 (64.7)	149 (38.5)	
Education level of person filling out the questionnaire			0.38			0.82
Junior high school	5 (2.4)	4 (3.0)		5 (1.9)	9 (2.3)	
Senior high school	40 (18.6)	36 (26.4)		58 (22.4)	103 (26.6)	
College and above	171 (79.0)	96 (70.6)		196 (75.7)	149 (71.1)	
MS score of plaque			0.01*			< 0.001**
0/1 (CFU < 10^5^)	153 (68.6)	78 (56.5)		228 (85.7)	273 (68.2)	
2/3 (CFU > 10^5^)	70 (31.4)	60 (43.5)		28 (14.3)	127 (31.8)	
MS score of saliva			0.28			< 0.001**
0/1 (CFU < 10^5^)	202 (90.6)	120 (87.0)		163 (61.2)	144 (36.0)	
2/3 (CFU > 10^5^)	21 (9.4)	18 (13.0)		103 (38.8)	256 (64.0)	
Caries experience at baseline						< 0.001**
Yes				43 (16.2)	267 (65.9)	
No				223 (83.8)	138 (34.1)	

*CF group* caries free at baseline, *MS* mutans streptococci

*Significant at *P* < 0.05; **Significant at *P* < 0.001

### Caries-free group

**Fig. 1:**
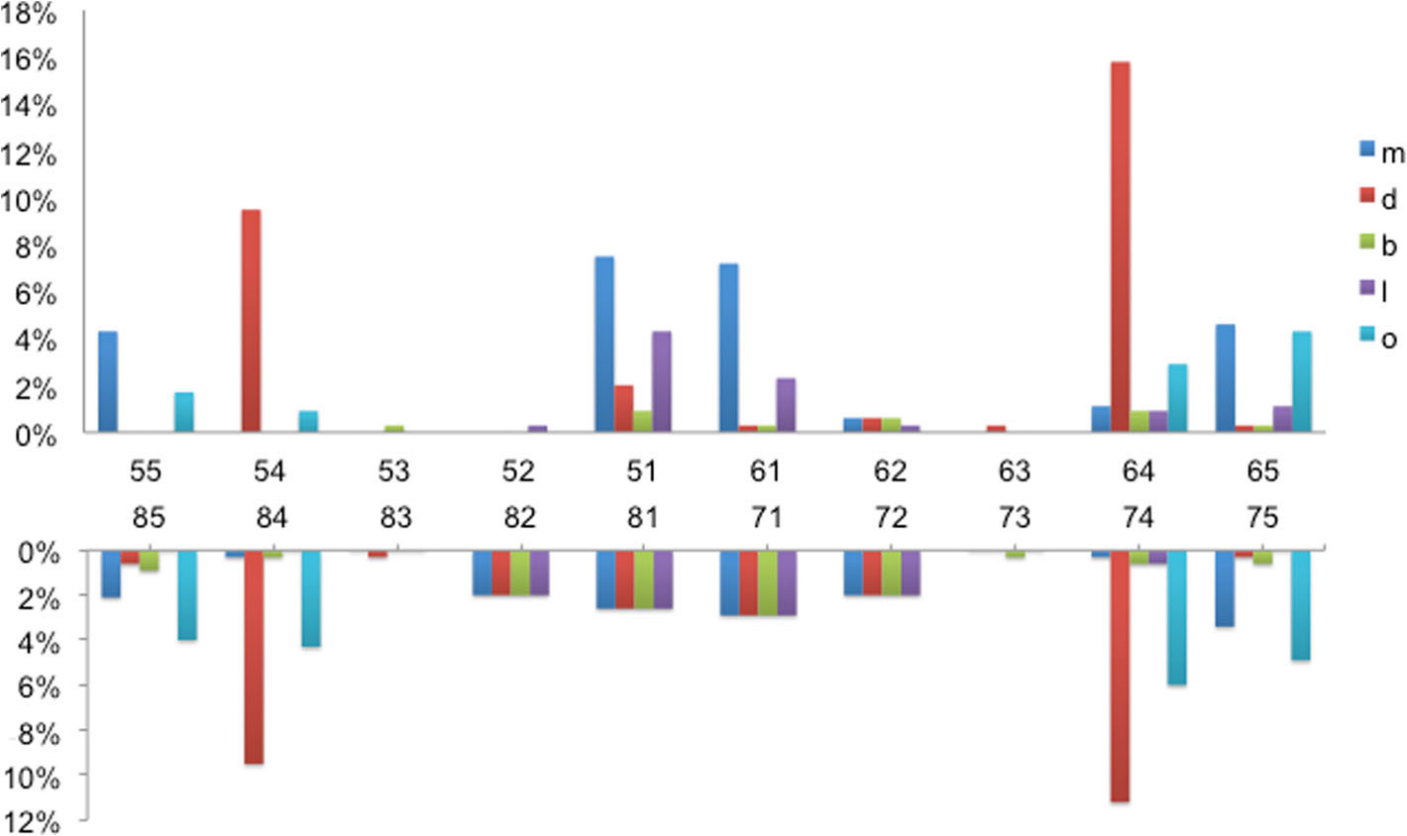
Proportion of tooth surfaces of new caries among caries-free children at 1-year follow-up. m: mesial surface; d: distal surface; b: buccal surface; l: lingual surface; o: occlusal surface

Among the 361 children in the caries-free group, 38.2% (138) children developed caries, and the mean dmfs score was 1.25 (±2.47) at 1 year. Distribution of new caries at the tooth surface level among these caries-free children at baseline is presented in Fig. 1. The tooth surface most sensitive to decay was the distal surface of primary upper left first molar (tooth 64:15.8%), followed by distal surfaces of other primary first molars (tooth 74: 11.2%, tooth 54 and 84: 9.5%) and mesial surfaces of primary maxillary central incisors (tooth 51:7.5%, tooth 61:7.2%).

Univariate analyses revealed that PMS level (*P* = 0.02) was the only significant risk factor, with an odds ratio (OR) of 1.68 (95% confidence interval [CI]: 1.08–2.61). Caries incidence and caries increment at 1-year follow-up were significantly higher in the high PMS score group (scored 2 and 3) than the low mutans streptococci score group (scored 0 and 1) (Table 2) (chi-square test, *t* test, *P* < 0.001).

**Table 2:** Caries incidence and increment among children with different PMS scores in the CF group

PMS score	N	With new caries (%) (Δdmft> 0)	△dmfs (mean ± SD)
0/1	231	78 (33.8)	0.90 ± 1.89
2/3	130	60 (46.2)	1.87 ± 3.17
		*P* = 0.02*	*P* < 0.001**

*PMS* plaque mutans streptococci at baseline, *CF group* caries free children at baseline, *dmfs* decayed, missing, or filled surfaces, △*dmfs* newly decayed, missing, or filled surfaces at the 1-year follow-up; △*dmft* newly decayed, missing, or filled teeth at the 1-year follow-up; *PMS* plaque mutans streptococci

*Significant at *P* < 0.05; **Significant at *P* < 0.001

### All participants

For all participants, eight variables were associated with caries increment by univariate analyses presented in Table 1: using nursing bottle or not at present (*P* = 0.01), frequency of snack consumption (*P* = 0.04), frequency of tooth brushing (*P* = 0.01), Toothpaste containing fluoride (*P* = 0.03), dental visit history (*P* < 0.001), PMS level (*P* < 0.001), SMS level (*P* < 0.001), and caries experience at baseline (*P* < 0.001). When these eight significant variables were analyzed together by using negative binomial regression, caries experience at baseline (*P* < 0.001) and PMS level (*P* < 0.001) were significant risk factors for new caries (Table 3). Children with high PMS levels (scored 2 and 3) had 1.69 (95% CI: 1.32–2.23) greater odds of developing new caries than those who had a low level of infection (scored 0 and l). Moreover, children with caries experience at baseline had 2.80 (95% CI, 2.20–3.56) greater odds of being at risk of developing new caries compared with children without any caries experience. Furthermore, the high PMS score group consisted of a significantly greater proportion of children who developed new caries compared with the low mutans streptococci score group (Table 4).

**Table 3:** Results of negative binomial regression for caries incidence at the 1-year follow-up among preschool children in Beijing

Variables	Negative binomial coefficients (95% CI)	Wald χ2	P
Intercept	1.80 (0.85, 2.75)	13.75	< 0.001**
Caries experience at baseline			
Yes	–	–	–
No	−1.03 (− 1.27, − 0.79)	70.03	< 0.001**
Using nursing bottle or not at present			
Yes	− 0.08 (− 0.35, 0.19)	0.35	0.56
No	–	–	–
Frequency of snack consumption			
Never or seldom	−0.25 (− 1.03, 0.52)	0.41	0.52
Sometimes (< 1 time/day)	−0.21 (− 0.89,0.48)	0.34	0.56
1–2 times/day	0.13 (−0.54,0.79)	0.14	0.70
3–4 times/day	0.25 (−0.44,0.94)	0.50	0.48
≥ 5 times/day	–	–	–
Frequency of tooth brushing			
Twice daily	−0.22 (− 1.12, 0.67)	0.24	0.63
Once daily	−0.07 (− 0.96, 0.83)	0.02	0.88
Less than once daily	−0.57 (− 1.51, 0.35)	1.47	0.23
Never	–	–	–
Toothpaste containing fluoride			
Yes	0.42 (− 0.16,1.00)	1.96	0.16
No	0.49 (− 0.09,1.07)	2.75	0.10
Not sure	0.56 (− 0.03,1.14)	3.44	0.06
Not having used toothpaste	–	–	–
Dental visit history			
Yes	0.16 (− 0.08, 0.40)	1.76	0.18
No	–	–	–
MS score in plaque			
0/1 (CFU < 10^5^)	−0.53 (− 0.79, − 0.27)	16.25	< 0.001**
2/3 (CFU > 10^5^)	–	–	–
MS score in saliva			
0/1 (CFU < 10^5^)	−0.12 (− 0.40, 0.15)	0.47	0.49
2/3 (CFU > 10^5^)	–	–	–

*CI* confidence interval, *MS* mutans streptococci

*Significant at *P* < 0.05; **Significant at *P* < 0.001

**Table 4:** Caries status at baseline and 1-year follow-up for children with different PMS scores

PMS score	N	Prevalence at baseline (%)	dmfs at baseline (mean ± SD)	With new caries (%) (Δdmfs> 0)	△dmfs (mean ± SD)
0/1	320	27.8	1.41 ± 3.53	45.0	1.51 ± 2.73
2/3	387	66.4	7.04 ± 9.66	66.1	3.59 ± 4.97
		*P* < 0.001**	*P* < 0.001**	*P* < 0.001**	*P* < 0.001**

*PMS* plaque mutans streptococci at baseline

*dmfs* decayed, missing, or filled surfaces, △*dmfs* newly decayed, missing, or filled surfaces at the 1-year follow-up, *PMS* plaque mutans streptococci

**Significant at *P* < 0.001

## Discussion

This prospective cohort study evaluated several factors related to 1-year development of ECC among 3- and 4-year-old children in Beijing. Statistics analysis identified the microbiological factor: a strong positive association was noted between the development of ECC and PMS levels among caries-free children at baseline and among all participants. These results were concordant with previous studies reporting that caries-susceptible individuals could be identified based on the correlation between presence of mutans streptococci and caries incidence [15–17]. Children with PMS scores 2 and 3 were more likely to develop new dental caries than those with PMS scores 0 and 1; this is in line with Seki’s observation [18] that high PMS score (2 or 3) was a risk factor for ECC (OR = 12.59, 95% CI: 3.18–67.08).

In the present study, the levels of mutans streptococci in plaque sampled from four smooth surfaces were tested using Dentocult SM strip test. This chair side method can be conveniently applied, and the scores can be easily evaluated according to the manufacturer’s instructions. The results revealed that it was practical to use Dentocult SM strips to identify caries risk among preschool children in Beijing. If their PMS score was 2 or 3 by using Dentocult SM strips, even if they are caries free, children could be thought to be at a high risk for developing ECC. Early intervention for mutans streptococci is suggested as soon as possible, including parental involvement along with behavioral and antimicrobial approaches for long-term caries prevention.

Both saliva and plaque were used to estimate mutans streptococci infection in this study. The association between dental caries increment and SMS levels, as observed in univariate analyses, disappeared when multivariate analyses were conducted. Although Nanda’s study reported a direct and strong correlation between the SMS and PMS levels and caries [19], we only detected a stronger correlation between PMS levels and development of ECC. These observations are in line with those reported in Seki’s research [18]. Theoretically, dental plaque is more appropriate for estimating mutants streptococci infection because tooth surfaces are the natural habitat of mutans streptococci [20]. Mutans streptococci in saliva are thought to be well below than their presence in dental plaque, and hence, SMS levels are considered to have an inferior correlation to caries than PMS levels. In fact, a previous study verified that the density of mutans streptococcus in saliva is lower than that in dental plaque [21]; saliva without paraffin stimulation was sampled in order to secure co-operation of the participating preschool children. Of note, non-stimulated saliva could replace stimulated saliva when Dentocult SM strips were used for preschool children [18, 21].

Besides PMS levels, the other risk factor identified in this study was caries experience. Given the multifactorial etiology of caries, studies have most commonly reported or reviewed a combination of mutans streptococci and caries experience as the best predictors of future caries incidence [22–24]. Although previous studies have demonstrated reduction of SMS levels after treatment of tooth decay, the relapse rate of ECC was still high and rapid [25, 26]. These remind guardians and pediatricians that dental cavities should be treated as early as possible, and appropriate interventions aimed at reducing mutans streptococci should be implemented to arrest recurrent caries.

Among caries-free children at baseline in our study, new visible caries developed most frequently in the distal surfaces of primary first molars, according with a similar study in China [27]. Of note, the contact areas of primary molars are broad and tight; such areas are ideal sites for carbohydrate residue and mutans streptococci and are tough to clean. Moreover, Leroy found that among 3–5-year-old high-risk children, new caries was mostly distributed in the distal and occlusal surfaces of primary first molars [28]; clinical significance stated that guardians should be instructed to use dental floss for their children, and caries-susceptible surfaces should be carefully inspected during clinical examination. Certain studies have shown that specific cariogenic bacteria were associated with development of different types of caries, such as *Streptococcus sobrinus* with smooth surface caries [29] and *Streptococcus mutans* with proximal caries [30]; however, more extensive researches are warranted to explore this proposed correlation between caries type and mutans streptococci. Neves et al. have found a low prevalence or even an absence of *Streptococcus mutans* in dentinal caries lesions [31], which verified the complexity between bacteria and caries.

In the present study, a negative correlation was identified between ECC and children’s dietary habits (e.g., the frequency of snack consumption) and oral hygiene behavior; the primary reason for this correlation may be that these are nonbiological factors. Although these were significant in univariate analysis, the significance faded when the direct biological factor—mutans streptococci—was analyzed simultaneously in multivariate analysis. A previous review confirmed that bacterial acquisition (primarily mutans streptococci) was mediated by maternal factors, oral health behavior and practices, and feeding habits [32].

Being a prospective cohort study including children who were 3- and 4-year old at baseline, the findings provides valuable insights into the risk factors of ECC in Beijing. With regard to limitations, the sample was not randomly selected (sample size and sampling method were described in the cross-sectional results presented in a previously published article) [14]. However, when the caries prevalence at baseline (49.3%) was compared with that (46.6%) of the same-aged children in a large-scale oral health survey conducted in Beijing during the similar period [33], no significant differences were detected. In the present study, the diagnostic criteria for caries were in accordance with the WHO criteria rather than the International Caries Detection and Assessment System (ICDAS), and no radiographs were used. These may lead to under or over estimation of the caries status and the development of caries due to neglect of early noncavitated lesions and/or white spots. However, at present, the WHO criteria are widely applied in China.

## Conclusion

This prospective cohort study provides a longitudinal assessment of the relationships between ECC and differently related factors. It confirmed the risk factors that affect ECC development. For caries-free children, high PMS levels (scored 2 and 3 by Dentocult SM strips) was the only significant risk factor, while for all children, besides high PMS levels, previous caries experience was the other significant risk factor. New tooth decay was most frequently distributed in the distal surfaces of primary first molars. The Dentocult SM strip test was a practical method to test the levels of mutans streptococci in both plaque and non-stimulated saliva of preschool children, and the former was superior at indicating the risk of ECC.

## Abbreviations

CFU: Colony forming units
CI: Confidence interval
CPI: Community periodontal index
ECC: Early childhood caries
ICDAS: International Caries Detection and Assessment System
OR: Odds ratio
PMS: Plaque mutans streptococci
SMS: Saliva mutans streptococci
